# Contrast Sensitivity after Zyoptix Tissue Saving LASIK and Standard LASIK for Myopia with 6-Month Followup

**DOI:** 10.1155/2011/839371

**Published:** 2011-03-16

**Authors:** Li-Quan Zhao, Huang Zhu

**Affiliations:** Department of Ophthalmology, School of Medicine, Xinhua Hospital Affiliated to Shanghai Jiao Tong University, 1665 Kongjiang Road, Shanghai 200092, China

## Abstract

This control-matched comparative study evaluated changes in contrast sensitivity after Zyoptix tissue-saving (TS) LASIK and Planoscan standard LASIK (Technolas 217z, Bausch & Lomb) for myopia 6 months postoperatively. 102 TS LASIK-treated eyes were matched with 102 standard LASIK-treated eyes (divided into low, moderate, and high groups). There were no significant differences in refraction outcomes between the groups postoperatively. In high group, a significant reduction in contrast sensitivity after TS LASIK was found at high spatial frequencies (*P* < .05) under photopic conditions and at middle to high spatial frequencies (*P* < .05) under mesopic conditions. And significant reduction was also found in standard LASIK at high spatial frequency (*P* < .05) under mesopic conditions. The reduction was significantly lower in TS LASIK than that in standard LASIK at high spatial frequencies (*P* < .05) under mesopic conditions. TS LASIK was prone to reduce mesopic contrast sensitivity of high myopia at high spatial frequencies.

## 1. Introduction

Laser in situ keratomileusis (LASIK) is an increasingly accepted surgery for myopia or compound myopic astigmatism [1, 2]. An optimal refractive surgery should need larger optical ablated zone avoiding night vision problems such as glare and halos for patients with larger pupil diameter, and less ablation of corneal tissue is needed for the same amount of dioptric correction [3–6]. But in order to remain an adequate residual stromal bed thickness, the patients with thinner central corneal thickness or higher myopia have to accept the LASEK surgery with some complications [7].

 Since the basic concept of Zyoptix tissue saving ablations in excimer corneal refractive surgery was introduced, more and more patients with thinner cornea or high myopia underwent this surgery. The Zyoptix tissue saving may reduce the amount of ablated corneal tissue intraoperatively. The basic concept of tissue saving LASIK includes measurement of the K value (corneal radius of curvature) with topography such as Orbscan II and transfer of the actual K value into a precise ablation pattern to be performed by 1- and 2-mm laser spot sizes. And an additional nonadjustable blend zone surrounding the adjustable optical zone is set to smaller than Planoscan platform.

Laser instruments differ in delivery platform, software design, ablation profile, and treatment zones and treat varying types of HOA, which could have a measurable impact on visual performance [8]. Hence, the outcomes of individual platforms need to be investigated to determine whether tissue-saving ablation is clinically advantageous compared with standard ablation. The purpose of this retrospective control-matched study was to compare the 6-month postoperative contrast sensitivity of standard LASIK (PlanoScan, Bausch & Lomb, Rochester, NY) and Zyoptix tissue saving LASIK (Zyoptix, Bausch & Lomb) using a scanning-spot 217z excimer laser (Bausch & Lomb) for low to high myopia.

## 2. Subjects and Methods

### 2.1. Study Design

The study was approved by the hospital's institutional review board. The two surgical techniques of Zyoptix tissue saving and standard LASIK were simultaneously performed in our hospital. Informed consent was obtained from all patients after a thorough explanation of the two procedures and their potential benefits and risks. Patients voluntarily selected their preferred surgical technique according to the personal refractive error and the corneal thickness. The charts of 2508 eyes that underwent Zyoptix tissue saving or standard LASIK were reviewed. Our database was compiled by excluding all eyes that had a preoperative manifest sphere greater than −9.00 diopters (D), hyperopic sphere, cylinder of more than −1.50 D, preoperative best-corrected visual acuity (BCVA) of 0.9 or worse, lost one or more lines of BCVA postoperatively, and those aimed for near correction. Patients with intraoperative or postoperative complications such as flap striae, epithelial ingrowths, diffuse lamellar keratitis, or infection were excluded. Each tissue-saving LASIK-treated eye was matched to a standard LASIK-treated eye using specific criteria. These criteria were (1) same preoperative sphere and cylinder, (2) difference within one line of BSCVA, (3) 6.0 mm optical zone, (4) nearest age and (5) completed a 6-month followup. After fulfilling all of the aforementioned criteria, two hundred and four eyes remained, among which 102 eyes had tissue-saving LASIK and 102 had standard LASIK. Each tissue-saving LASIK-treated eye was matched with a single standard LASIK-treated eye from the patient. The eyes were classified into three subgroups according to the sphere; Group 1: less than −3.00 D, Group 2: between −3.10 and −6.00 D, and Group 3: more than −6.10 D.

### 2.2. Examination

Preoperatively, all patients had a complete ophthalmic examination including uncorrected visual acuity (UCVA) and best-corrected visual acuity (BCVA) using a Snellen chart, Nidek auto chart projector ACP 8, and including manifest and cycloplegic refractions. Manifest and cycloplegic refractions, ocular dominance, corneal topography (Orbscan II z, version 3.10.31, Bausch & Lomb.), applanation tonometry, slitlamp evaluation of the anterior segment, and ophthalmoscopy were performed. Pupil size was measured under scotopic conditions with the ARK-730A pupillometer (NIDEK CO., Ltd, Japan) by taking the mean of three measurements. The corneal radiuses of curvature were measured by the Orbscan II topographer, which operates on the principle that light rays. Three Orbscan II maps were taken, and the one with the least eye movements was used. The maximum movements considered acceptable were 200 *μ*m. 

Functional acuity contrast sensitivity (F.A.C.T.) was measured before and six months after surgery using the Optec 6500 vision testing system (Stereo Optical Co, Inc, Chicago, Illinois, USA) with best correction under photopic condition (target luminance value of 85 cd/m^2^) without glare, mesopic (target luminance value of 3 cd/m^2^) without glare. Contrast sensitivity was tested at the spatial frequencies of 1.5, 3, 6, 12, and 18 cycles per degree (cpd). All contrast sensitivity data were transformed to logarithmic units and log contrast sensitivity values were compared before and after surgery in each group.

All examinations were performed by the same person using the same procedure and equipment.

### 2.3. Surgical Technique

All patients were operated by a single surgeon (Huang Zhu). The LASIK procedures were performed using topical anesthesia of oxybuprocaine 0.4% without preservatives. A flap was created with a superior hinge with a Hansatome microkeratome (Bausch & Lomb). The ablation was performed using the 193 nm 217z scanning-spot excimer laser system with a combined 2.0 mm and 1.0 mm spot in the Zyoptix tissue saving group, and a Zyoptix treatment card was inserted. The Standard group was treated with a 2.0 mm scanning spot. A residual stromal bed of 280 *μ*m or more was left in all eyes. The optical zone was set at 6.0 mm. The eye track was kept on during laser ablation. The eye tracker was kept on during laser ablation. The Standard LASIK was used for the Planoscan software, and Zyoptix tissue-saving LASIK was used for the Zyoptix software inputting customized *K* value. Before each treatment, the laser was calibrated by a fluence test and the eye-tracking system was tested. The radiant exposure was 0.2 J/cm^2^ in the treatment plane, and the repetition frequency of the laser was 120 Hz.

### 2.4. Data Analysis

Results of subjective refraction were collected and converted to spherical equivalent (SE) values for analysis. Each *K* value of 5 mm optical zone was analyzed using the Orbscan II software provided by the manufacturer. Tissue ablation depth was determined by entering the desired correction into the laser and using the laser algorithm (Zyoptix, version 4.21). All contrast sensitivity data were transformed to logarithmic units and log contrast sensitivity values for analysis.

 All data are recorded as the mean ± SD. Statistical analysis was performed using SPSS 13.0 software. Paired-samples *t* tests were applied. A *P* value of less than .05 was considered statistically significant.

## 3. Results

### 3.1. Refraction Outcomes

Table 1 shows baseline patient characteristics for six subgroups. No statistically significant differences were noted between the tissue-saving and standard LASIK with respect to age, gender, spherical equivalent refraction, preoperative BCVA. In low and moderate myopia groups, there were no statistically significant differences in keratometry (*K* value) and pachymetry. In high myopia group, the pachymetry was significantly thinner (*P* < .001) and keratometry was significantly higher (*P* = .001) in TS group than those in standard group. In moderate and high myopia groups, the attempted spherical equivalent refraction was significantly higher in TS group than those in standard group. In all groups, the tissue ablation depth were significantly less in TS group than those in standard group and cornea tissues were preserved 18.32%, 18.34%, and 21.42%, respectively.

 Six months postoperatively, there were no statistically significant differences in UCVA, BCVA, and residual SE, and the proportion of SE was within ±0.50 D and ±1.00 D.

### 3.2. Contrast Sensitivity

In three groups, there were no significant differences in preoperative contrast sensitivity at any spatial frequencies under photopic and mesopic conditions between the TS LASIK-treated eyes and standard LASIK-treated eyes.

In low and moderate myopia group, contrast sensitivity at all spatial frequencies for eyes after TS LASIK and standard LASIK did not significantly differ from preoperative values under the photopic and mesopic conditions and there were no significant differences in postoperative contrast sensitivity at all spatial frequencies between two groups.

In high myopia group, a statistically significant reduction in contrast sensitivity after TS LASIK was found at high spatial frequencies (*P* < .05 for 12 and 18 cpd) under photopic conditions and at middle and high spatial frequencies (*P* < .05 for 6, 12, and 18 cpd) under mesopic conditions. And significant reductions were also found in standard LASIK at high spatial frequency (*P* < .05 for 18 cpd) under mesopic conditions. The reductions were significant lower in TS LASIK than that in standard LASIK at high spatial frequencies (*P* < .05 for 12 to 18 cpd) under mesopic conditions.

## 4. Discussion

Zyoptix tissue-saving LASIK and PlanoScan standard LASIK are both efficient refractive surgery techniques for patients with low to high myopia. Our findings are similar to a recent study comparing the Bausch & Lomb Zyoptix (tissue-saving ablation) treatment protocol to the Technolas PlanoScan (conventional ablation) treatment, which found no difference in refraction or visual outcomes [9]. Because the tissue-saving LASIK had more undercorrections in the moderate to high myopia, the amount of attempted corrections were more than those of the standard treatment for same moderate or high myopia. The achieved corrections were the same. The postoperative residual refractive errors were similar. Although our control-matched comparative study showed that both Zyoptix tissue-saving LASIK and standard LASIK achieved similar excellent results of safety, efficiency, and predictability for myopia less than −9.00 D, importantly, our study focuses on researching two different excimer lasers of software design and ablation profile impact on contrast sensitivity under photopic and mesopic conditions. 

In the present study, preoperative contrast sensitivity values were similar in both groups. Under mesopic conditions, contrast sensitivity values reduced postoperatively in both groups, especially for high myopia at high spatial frequecies. This was an expected result, because LASIK has been reported to induce temporary or long-term reduction in low-contrast visual acuity or contrast sensitivity, particularly at higher spatial frequencies under mesopic conditions [10, 11]. And Bailey's research revealed that low-contrast visual acuity of patients with myopia up to −6 diopters were significantly worse than low myopes [12]. The greater the correction or the more corneal tissue ablated, the greater the initial decrease in contrast sensitivity [13–15].

We also found that mesopic contrast sensitivity values after tissue-saving LASIK significantly deteriorated not only across a wider range (middle to high) of spatial frequencies for high myopia, but also were significantly worse at high spatial frequencies for high myopia than that after the standard LASIK 6 months postoperatively. Besides mesopic contrast sensitivity, photopic contrast sensitivity after TS LASIK also significantly declined at high spatial frequencies in high myopia.

This may reflect that the differences of contrast sensitivity were not determined by the ablation depth, because the eyes with removal of less corneal tissue in the tissue-saving group theoretically gained better contrast sensitivity for same myopia. Obviously, there was a significant correlation of ablation profile (especially optical zone and blend zone) between the two nomograms with different changes in mesopic contrast sensitivity by two different LASIK procedures. 

Previous studies have proved that higher order aberrations induced by LASIK are thought to be responsible for mesopic visual symptoms such as halo, glare, and starburst [16]. Yamane demonstrated that induced changes in the contrast sensitivity function significantly correlated with increases in ocular higher order aberrations, particularly spherical aberrations [17]. They also reported that increases in higher order wavefront aberrations showed a significant positive correlation with the amount of myopic correction. These results indicate that the larger the attempted corrections, the greater the amount of central corneal tissue that is removed by laser ablation. LASIK-induced transformation of corneal asphericity from prolate to oblate causes coma, astigmatism, distortion, and spherical aberration [18]. Besides the amount of ablated tissue, the effect of increased spherical aberration after LASIK for treatment of myopia may be partially dependent on the relationship of the pupil size and ablation zone. Clinical findings of increasing incidence of glare, halo, and disturbance of night vision were partially attributed to the smaller ablation diameter and larger pupil diameter. Mok's research revealed that the amount of LASIK-induced higher-order aberrations appeared to be associated with optical zone diameter [6]. A larger optical zone diameter may theoretically improve visual quality after LASIK as it reduces the difference between the size of the ablation zones and the enlarged scotopic pupil, which may lead to less higher order aberrations. Mesopic testing results in a larger physiologic pupil size. As higher order aberrations increase with larger pupils, it would be intuitive that testing under these reduced light levels would better reflect reduced optical quality due to higher order aberrations. Pepose showed that mesopic low contrast acuity was more affected by subtle variations in higher order aberrations than photopic high contrast acuity [19]. Smaller zones lead to an increased pupil-to-clear zone ratio during scotopic conditions and result in higher-order spherical aberrations and patient dissatisfaction from increased glare and halos [20, 21]. The optimized postconventional LASIK vision quality might be due to the increased clearance zone with the larger optical zone diameter. 

In the present study, the optical zone of selected eyes was similar to adjust to 6.0 mm. As Kirwan described, an additional nonadjustable blend zone of 3 mm diameter surrounds the optical zone in Planoscan treatments, but the blend zone is considerably smaller at 0.875 mm in Zyoptix treatments [9]. He concluded that the smaller blend zone incorporated into tissue-sparing treatments may be responsible for the greater postoperative increase in HOAs compared with conventional Planoscan treatments. Previous studies showed that a larger effective treatment zone with a smoother transition zone between the central and peripheral cornea may aid in the prevention of postoperative symptoms such as halos and starbursts with large scotopic pupils [21, 22]. Hence, in this case, the deteriorated contrast sensitivity after tissue-saving LASIK was attributable to the smaller blend zone. And this result was consistent with the conclusion that the smaller blend zone of tissue-saving LASIK induced greater increase in HOA. In Kirwan's study, the factors were excluded that the ablation depth, optical zone, and the scotopic pupil size were similar. Although the ablation depth (less in tissue-saving LASIK) were not control-matched in the present study, the result was still convincing.

## 5. Conclusion

In summary, the present study showed that Zyoptix tissue-saving LASIK may be a safe and effective procedure that improves the visual performance. A most important advantage of this platform is the sparing of tissue ablation depth in the range of 20–25%, which removes less tissue than standard laser treatments. It is convenient to high myopia or thinner cornea for thicker residual stromal bed. But the ablation algorithms of blend zone affect the postoperative vision quality, particularly mesopic contrast sensitivity. When deciding on a procedure for tissue-saving LASIK, the structural safety and vision quality of the eye would have to be taken into account.

## Figures and Tables

**Table 1:** Baseline characteristics of 102 matched eyes that underwent tissue-saving LASIK or standard LASIK treatment for myopia.

Groups	Group 1 (sphere *⩽* −3D)			Group 2 (−3D < sphere ⩽ −6D)			Group 3 (sphere >−6D)		
	Standard-	TS-	*P*	Standard-	TS-	*P*	Standard-	TS-	*P*
No. (M/F)	26 (17/9)	26 (15/11)		44 (15/29)	44 (24/20)		32 (9/23)	32 (12/20)	
Age (years)	23.80 ± 6.13	28.04 ± 7.73	.051	27.10 ± 6.26	25.02 ± 6.45	.064	25.16 ± 5.96	25.34 ± 6.26	.906
Sphere (D)	2.58 ± 0.41	2.58 ± 0.41		4.68 ± 0.77	4.68 ± 0.77		7.02 ± 0.63	7.02 ± 0.63	
Cylinder (D)	0.46 ± 0.31	0.46 ± 0.31		0.60 ± 0.35	0.60 ± 0.35		0.52 ± 0.38	0.52 ± 0.38	
Pachymetry (*μ*m)	544.12 ± 27.75	540.04 ± 29.35	.648	561.73 ± 30.12	552.05 ± 33.88	.168	572.65 ± 28.87	542.97 ± 23.47	<.001
*K*-value	42.77 ± 0.79	42.53 ± 0.92	.266	42.87 ± 1.24	42.45 ± 1.12	.103	42.38 ± 1.13	43.30 ± 1.13	.001
Attempted SE (D)	−3.45 ± 0.43	−3.46 ± 0.39	.916	−5.55 ± 0.78	−5.76 ± 0.83	<.001	−7.85 ± 0.55	−8.03 ± 0.68	.004
Ablation depth (*μ*m)	64.19 ± 8.00	52.23 ± 5.54	<.001	102.25 ± 14.55	83.50 ± 11.04	<.001	141.81 ± 10.59	111.44 ± 9.50	<.001
Pre-BCVA	1.25 ± 0.15	1.18 ± 0.14	.114	1.25 ± 0.16	1.20 ± 0.14	.103	1.19 ± 0.13	1.13 ± 0.17	.094
Post-UCVA	1.21 ± 0.16	1.17 ± 0.13	.399	1.19 ± 0.19	1.16 ± 0.17	.433	1.10 ± 0.14	1.08 ± 0.15	.495
Post-BCVA	1.40 ± 0.14	1.36 ± 0.15	.212	1.33 ± 0.15	1.34 ± 0.16	.999	1.29 ± 0.16	1.22 ± 0.17	.102
Post-SE(D)	−0.21 ± 0.27	−0.30 ± 0.26	.128	−0.14 ± 0.35	−0.28 ± 0.35	.101	−0.30 ± 0.38	−0.31 ± 0.45	.924
SE with ± 0.5D(%)	96.2 (25/26)	92.3 (24/26)		88.6 (39/44)	84.1 (37/44)		75.0 (24/32)	78.1 (25/32)	
SE with ± 1.0D(%)	100 (26/26)	100 (26/26)		100 (44/44)	100 (44/44)		100 (32/32)	100 (32/32)	
Scotopic pupil size (mm)	6.58 ± 0.68	6.56 ± 0.73	.935	6.55 ± 0.79	6.59 ± 0.74	.740	6.56 ± 0.79	6.59 ± 0.64	.781

LASIK: laser in situ keratomileusis; D: diopters; TS-: tissue saving; m: male; f: female; SE: spherical equivalent; BCVA: best-corrected visual acuity; UCVA: uncorrected visual acuity; pre-: preoperative; post-: postoperative; *P* value was calculated using paired samples *t* test.

**Table 2:** Pre- and postoperative contrast sensitivity in Group 1 (sphere *⩽*−3D) underwent tissue-saving LASIK or standard LASIK.

*N* = 26		Photopic					Mesopic				
	Spatial frequency	1.5	3	6	12	18	1.5	3	6	12	18

Standard-	Pre-	1.81	1.94	1.78	1.26	0.81	1.77	1.94	1.68	1.24	0.79
	Post-	1.80	1.96	1.81	1.27	0.83	1.79	1.97	1.75	1.24	0.80
Ts-	Pre-	1.80	1.95	1.82	1.28	0.80	1.76	1.93	1.68	1.20	0.78
	Post-	1.83	1.96	1.80	1.28	0.82	1.80	1.97	1.74	1.24	0.81

	*P *(*t* test)										

Standard-	Pre- versus Post-	0.782	0.638	0.343	0.852	0.381	0.187	0.291	0.076	0.983	0.713
TS-	Pre- versus Post-	0.247	0.820	0.428	0.953	0.375	0.186	0.073	0.109	0.106	0.124
Pre-	Standard- versus TS-	0.799	0.828	0.475	0.580	0.835	0.971	0.702	0.951	0.297	0.630
Post-	Standard- versus TS-	0.202	0.999	0.683	0.623	0.825	0.745	0.978	0.773	0.886	0.755

LASIK: laser in situ keratomileusis; D: diopters; TS-: tissue saving; pre-: preoperative; post-: postoperative.

**Table 3:** Pre- and postoperative contrast sensitivity in Group 2 (−3D < sphere ⩽−6D) underwent tissue-saving LASIK or standard LASIK.

*N* = 44		Photopic					Mesopic				
	Spatial frequency	1.5	3	6	12	18	1.5	3	6	12	18

Standard-	Pre-	1.79	1.90	1.77	1.24	0.80	1.76	1.88	1.61	1.17	0.76
	Post-	1.77	1.91	1.71	1.21	0.77	1.78	1.90	1.65	1.15	0.74
TS-	Pre-	1.80	1.92	1.78	1.25	0.79	1.78	1.91	1.68	1.18	0.77
	Post-	1.79	1.91	1.74	1.22	0.76	1.78	1.93	1.65	1.16	0.75

	*P *(*t* test)										

Standard-	Pre- versus Post-	0.212	0.801	0.199	0.066	0.058	0.579	0.235	0.284	0.373	0.484
TS-	Pre- versus Post-	0.412	0.574	0.055	0.147	0.100	0.878	0.144	0.449	0.155	0.278
Pre-	Standard- versus TS-	0.700	0.458	0.888	0.926	0.487	0.579	0.125	0.129	0.661	0.434
Post-	Standard- versus TS-	0.538	0.876	0.592	0.631	0.808	0.874	0.146	0.948	0.678	0.686

LASIK: laser in situ keratomileusis; D: diopters; TS-: tissue saving; pre-: preoperative; post-: postoperative.

**Table 4:** Pre- and postoperative contrast sensitivity in Group 3 (sphere >−6D) underwent tissue-saving LASIK or standard LASIK.

*N* = 44		Photopic					Mesopic				
	Spatial frequency	1.5	3	6	12	18	1.5	3	6	12	18

Standard-	Pre-	1.81	1.95	1.72	1.24	0.77	1.76	1.91	1.69	1.20	0.75
	Post-	1.78	1.90	1.67	1.19	0.76	1.75	1.93	1.63	1.17	0.71
TS-	Pre-	1.80	1.91	1.69	1.21	0.76	1.76	1.86	1.67	1.16	0.75
	Post-	1.76	1.87	1.64	1.11	0.69	1.74	1.88	1.57	1.09	0.65

	*P *(*t* test)										

Standard-	Pre- versus Post-	0.228	0.053	0.069	0.127	0.556	0.869	0.376	0.244	0.448	0.046
TS-	Pre- versus Post-	0.125	0.066	0.055	0.001	0.013	0.443	0.457	0.024	0.016	0.001
Pre-	Standard- versus TS-	0.891	0.358	0.547	0.386	0.629	0.998	0.250	0.763	0.208	0.796
Post-	Standard- versus TS-	0.614	0.171	0.414	0.010	0.011	0.683	0.097	0.289	0.034	0.031

LASIK: laser in situ keratomileusis; D: diopters; TS-: tissue saving; pre-: preoperative; post-: postoperative.
